# Laparoscopic versus open mesh repair of bilateral primary inguinal hernia: A three-armed Randomized controlled trial

**DOI:** 10.1016/j.amsu.2020.08.055

**Published:** 2020-09-09

**Authors:** M.M. Elmessiry, A.A. Gebaly

**Affiliations:** Department of Surgery, Alexandria Faculty of Medicine, Alexandria, Egypt

**Keywords:** Bilateral primary inguinal hernia, Laparoscopic TAPP, Open pre-pritoneal repair, Bilateral lichtenstein repair, Operative outcomes, Quality of life, Recurrence

## Abstract

**Purpose:**

The best approach for simultaneous repair of bilateral inguinal hernia is controversial. The aim of this study is to compare the outcomes after laparoscopic versus open mesh repair of bilateral primary inguinal hernia.

**Methods:**

this prospective study included 180 patients with bilateral primary inguinal hernia; randomized by sealed envelopes into 3 groups; each includes 60 patients. Group I treated by laparoscopic trans-abdominal pre-peritoneal (TAPP) repair using 2 separate meshes, Group II treated by open pre-peritoneal (PP) single mesh repair, while Group III treated by bilateral Lichtenstein repair.

**Results:**

In comparison to open PP and bilateral Lichtenstein repair, Laparoscopic TAPP repair had significantly longer operative time and superior early postoperative outcomes including significantly less postoperative pain, hospital stay, time till return to normal activity and to work. Chronic groin pain and mesh sensation was lower in Laparoscopic TAPP group with significantly higher satisfaction rate compared to open groups. No significant difference between study groups in 3 years recurrence rate.

**Conclusion:**

Simultaneous laparoscopic TAPP repair of uncomplicated primary bilateral inguinal hernia has superior early postoperative outcome, less chronic pain and higher patients' satisfaction rate compared to open approaches with similar low recurrence rate.

## Highlights

• Hernia Surgery Group recommended one stage mesh repair of bilateral primary inguinal hernia; however, the ideal approach is controversial.
• Laparoscopic and open repair of bilateral inguinal hernia were compared regarding early outcomes, quality of life ‎and recurrence rate.
• This study demonstrated that laparoscopic TAPP repair of bilateral primary inguinal hernia has superior early postoperative outcomes.
• Laparoscopic TAPP repair has less chronic pain and higher patients' satisfaction rate compared to open approaches with low recurrence rate.

## 1 Background

Many studies recommended one stage tension free mesh repair of bilateral inguinal hernia, however, there is a controversy regarding the ideal surgical technique [1,2,3]. The aim of this study was to compare the outcome of laparoscopic versus open repair of bilateral primary inguinal hernia. The primary endpoint is early operative outcomes including operative time, postoperative complications, hospital stay, postoperative pain, timing of return to normal activity and work. The secondary endpoint is intermediate term outcomes after 3 years including quality of life, hernia recurrence and patient's satisfaction.

## 2 Patients and methods

**Fig. 1:**
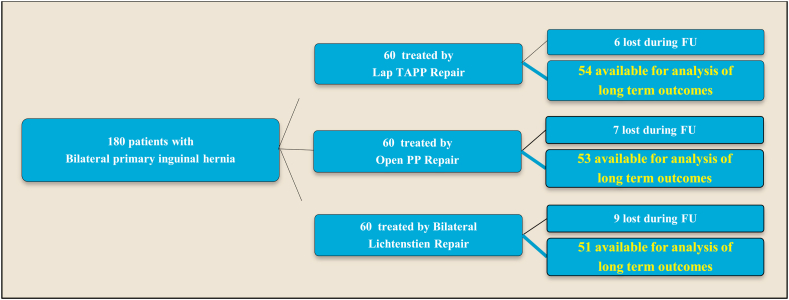
Consort FlowChart: Patients included in all groups until completeness of follow up& analysis.

This prospective randomized study included 180 consecutive patients with bilateral primary inguinal hernia managed by simultaneous bilateral repair at Alexandria University hospital between June 2014 and June 2017. Inclusion criteria were patients with painless uncomplicated primary bilateral inguinal hernias aged from 20 to 80 years. Exclusion criteria included immune compromised patients, chronic liver or renal disease, coagulopathy, high-risk patients unfit for major surgery (ASA III or IV), massive scrotal, recurrent, or complicated hernias, groin pain due to any other pathology and previous infra-umbilical surgery. Patients were randomized by sealed opaque envelopes containing computer generated random numbers into 3 groups, each includes 60 patients. Group I treated by laparoscopic trans-abdominal pre-peritoneal repair using 2 separate meshes fixed by endoscopic tackers (Lap TAPP), Group II treated by open pre-peritoneal single mesh repair with suture fixation (Open PP), while Group III treated by standard bilateral Lichtenstein repair (LICHT group). After proper patient counseling and taking informed consent, an envelope was opened and the patient was then offered the allocated hernia repair. European Hernia society (EHS) classification [4] was used to classify the hernia on both sides according to anatomical defect and size, where the size of the hernia orifice is registered as 1 (<1 finger), 2 ( > 1 and < 3 fingers) and 3 (,>3 fingers). For the anatomic localization; (L = lateral, M = medial, F = femoral). To avoid bias, all surgeries were performed by 2 surgeons experienced in both laparoscopic and open techniques of hernia repair and the results were recorded by a medical officer who was not involved in the surgery. Moreover, a standardized protocol was followed in all patients including urinary catheter insertion after induction of anesthesia for bladder decompression, monofilament polypropylene mesh used and fixed in all patients, 3rd generation cephalosporin given intravenously prior to induction of anesthesia and continued postoperatively till replaced by oral antibiotic upon discharge. Postoperative analgesia included intra muscular non-steroidal anti-inflammatory in the first post-operative day then replaced by oral only as needed. Early postoperative complications within 30 days after the surgery were recorded. Postoperative pain intensity was assessed 24 h and 7 days after surgery using pain visual analogue scale (VAS) with values ranging from 0 (no pain) to 10 (worst possible pain). Follow up was done for 3 years as outpatient clinic visits 1, 2, 4 weeks after surgery then every 3 months in the 1st year then every 6 months. 6 patients in Lap TAPP group, 7 patients in open PP group and 9 patients in Bilateral Lichtenstein group didn't complete the follow-up and were excluded as shown in (CONSORT) flow chart (Fig. 1). The follow up included clinical assessment and abdominal imaging by ultrasonography or CT if recurrence was suspected. Quality of life was assessed using Carolina comfort scale [5]; which assess degree of discomfort caused by Sensation of mesh, Pain and Movement limitations at different positions and activities with a total score ranging from 0 to 115. Patient satisfaction was assessed using 0 to 10 scale where: 9–10 means very satisfied, 7–8: satisfied, 5–6: neutral, 3–4: dissatisfied, 0–2: very dissatisfied. Taking into consideration all patients had no preoperative pain as patients with complicated hernia or groin pain due to any other cause were excluded, chronic postoperative groin pain was defined as groin pain related to surgery lasting for 3 months or more [6] The 3 groups were compared regarding: operative time, postoperative complications, postoperative pain, hospital stay, time to return to normal activity and work as well as Quality of life, 3 years-recurrence rate and patient's satisfaction.

## 3 Statistical analysis

Numerical data in both groups was expressed as mean ± standard deviation (SD) and compared using One-way analysis of variance while categorical data was expressed as percentages and compared using Chi-squared test. Logistic regression test was used to determine predictors of postoperative complications. Differences were considered significant at p < 0.05.

## 4 Results

Patients' age ranged from 34 to 80 years with no significant statistical difference between the 3 groups regarding patients’ characteristics, comorbidities, anatomical classification and size of hernia (Table 1).

**Table 1:** Patients and hernia characteristics.

		Bilateral TAPP (n = 54)		Open PP Repair (n = 53)		Bilateral Lichtenstein (n = 51)		X2/F	P
		No	%	No	%	No	%		
Gender	Male	36	66.70%	44	83.00%	36	3.974	3.974	0.137
	Female	18	33.30%	9	17.00%	15	3.974		
Age (mean ± SD) (Min – Max)		53.03 ± 11.23 38–80		53.73 ± 10.44 36–78		55.85 ± 11.91 39–79		1.356	0.262
Smoking	Non smoker	9	16.70%	6	11.30%	7	13.70%	0.640	0.726
	Smoker	45	83.30%	47	88.70%	44	86.30%		
BMI (mean ± SD) (Min – Max)		24.88 ± 3.88 18.79–34.10		24.97 ± 4.76 18.79–34.20		24.61 ± 3.82 18.70–33.80		0.266	0.767
Obesity	Non Obese	44	81.50%	38	71.70%	41	80.40%	1.767	0.413
	Obese	10	18.50%	15	28.30%	10	19.60%		
DM	No	46	85.20%	40	75.50%	42	82.40%	1.729	0.421
	Yes	8	14.80%	13	24.50%	9	17.60%		
ASA score	ASA 1 Normal healthy patient	38	70.40%	31	58.50%	36	70.60%	2.270	0.321
	ASA 2 Mild systemic disease	16	29.60%	22	41.50%	15	29.40%		
Hernia Type Classification	Bilateral Medial Hernia	28	51.90%	24	45.30%	24	47.10%	1.199	0.878
	One Medial and one Lateral Hernia	14	25.90%	14	26.40%	16	31.40%		
	Bilateral Lateral Hernia	12	22.20%	15	28.30%	11	21.60%		
Hernia Size Classification	Size 2 both sides	21	38.90%	21	39.60%	23	45.10%	0.955	0.917
	Size 2 one side & 3 other side	27	50.00%	24	45.30%	22	43.10%		
	Size 3 both sides	6	11.10%	8	15.10%	6	11.80%		

### 4.1 Operative data and early postoperative outcomes (Table 2)

74.5% of Bilateral Lichtenstein and 28.3% of open PP repair were done under spinal anesthesia, while all laparoscopic TAPP repairs were done under general anesthesia. Laparoscopic TAPP repair had significantly longer operative time; 94.3 ± 10.19 minutes, compared to 90.89 ± 8.13 minutes for open PP repair and 84.22 ± 3.73 for bilateral Lichtenstein repair (p < 0.001). Intraoperative bleeding happened in one patient (2.5%) in Lap TAPP and two patients (3.80%) in open PP repair group without significant difference. Incidence of early postoperative complications after Laparoscopic TAPP repair was only 5.6% which is significantly lower in comparison to 20.8% and 21.6% after open PP and bilateral Lichtenstein groups respectively; p 0.039. The incidence of urinary retention was 1.9% in Lap TAPP, compared to 9.4% in open PP repair and 3.9% in Bilateral Lichtenstein repair, p 0.182. Scrotal hematoma was reported in 1 cases (1.9%) in Lap TAPP group; compared to 7 (13.2%) in open PP and 5 (9.8%) in bilateral Lichtenstein group, p 0.09. Pelvic hematoma was detected only in 1 case (1.9%) in Lap TAPP group and 3 cases (5.7%) in open PP, (p 0.1247). One patient (1.9%) in Lap TAPP group developed port site seroma compared to 7 (13.2%) in open PP and 9 (17.6%) in bilateral Lichtenstein group (p0.026). Three patients; 2 in open PP and one in bilateral Lichtenstein group required seroma aspiration for evacuation, while the other cases responded to compression and anti-inflammatory medications. Four patients (7.5%) in open PP group and three patients (5.9%) in bilateral Lichtenstein group developed postoperative wound infection or dehiscence, while no patient in lap TAPP group reported port site infection (p 0.137). All of the cases responded to conservative treatment except one patient in the open PP group required wound drainage and debridement.

Laparoscopic TAPP repair had superior immediate postoperative outcomes including less postoperative pain scores, shorter duration of hospital stay, time till return to normal activity and to work (Table 2). The mean pain score by the visual analogue scale after the 1st 24 h was significantly less after Lap TAPP compared to open PP repair and Bilateral Lichtenstein repair (3.37 ± 0.71 compared to 4.81 ± 0.74 and 5.12 ± 1.69 respectively, P < 0.001). The same pattern was recorded after 7 days, the mean pain score was 1.81 ± 1.21 in Lap TAPP group, compared to 4.13 ± 0.88 and 3.18 ± 0.71 in open PP and Bilateral Lichtenstein groups respectively, P < 0.001. Hospital stay was significantly shorter in Lap TAPP group compared to open PP group and Bilateral Lichtenstein (1.11 ± 0.32 versus 1.77 ± 0.452 and 1.41 ± 0.50 days, P < 0.001). Lap TAPP group had a significantly faster Return to daily activity compared to open PP group and Bilateral Lichtenstein; (5.87 ± 0.97 compared to. 10.64 ± 0.96 and 12.10 ± 1.02 days respectively, P < 0.001). Moreover, Lap TAPP group had a significantly shorter time for Return to work compared to open PP group and Bilateral Lichtenstein (12.30 ± 1.47 vs. 19.85 ± 1.06 and 20.20 ± 1.79 days respectively, p 0.001).

**Table 2:** Operative data and early postoperative outcomes.

		Bilateral TAPP (n = 54)		Open PP Repair (n = 53)		Bilateral Lichtenstein (n = 51)		X2/F	P
		No	%	No	%	No	%		
Anesthesia	Spinal	0	0.00%	15	28.30%	38	74.50%	66.304	<0.001*
	General	54	100.00%	38	71.70%	13	25.50%		
Surgeron	Surgeron 1	34	63.00%	31	58.50%	29	56.90%	0.438	0.803
	Surgeron 2	20	37.00%	22	41.50%	22	43.10%		
Operative time (minutes): (mean ± SD) (Min – Max)		94.3 ± 10.19 82–118		90.89 ± 8.13 80–115		84.22 ± 3.73 78–90		22.28	<0.001*
Operative Bleeding	No	53	98.10%	51	96.20%	51	100.00%	2.051	0.370
	Yes	1	1.90%	2	3.80%	0	0.00%		
Pain score after 24 h (mean ± SD) (Min – Max)		3.37 ± 0.71 2–5		4.81 ± 0.74 3–6		5.12 ± 1.69 3–7		36.18	<0.001*
Pain scale (24 h)	No Pain (VAS 0)	0	0.00%	0	<0.001*	0	0.00%	36.655	0.001*
	Mild pain (VAS 1–3)	30	55.60%	1	<0.001*	18	35.30%		
	Mod pain (VAS 4–7)	24	44.40%	52	<0.001*	33	64.70%		
	Severe pain (VAS 8–10)	0	0.00%	0	<0.001*	0	0.00%		
Pain score after 7 days (mean ± SD) (Min – Max)		1.81 ± 1.21 0–4		4.13 ± 0.88 2–6		3.18 ± 0.71 2–5		78.35	<0.001*
Pain scale (7 days)	No Pain (VAS 0)	11	20.40%	0	<0.001*	0	0.00%	82.599	<0.001*
	Mild pain (VAS 1–3)	40	74.10%	10	18.90%	37	72.50%		
	Mod pain (VAS 4–7)	3	5.60%	43	81.10%	14	27.50%		
	Severe pain (VAS 8–10)	0	0.00%	0	0.00%	0	0.00%		
Early Postop Complications (3 m)	No	51	94.40%	42	79.20%	40	78.40%	6.506	0.039*
	Yes	3	5.60%	11	20.80%	11	21.60%		
Urine retention	No	53	98.10%	48	90.60%	49	96.10%	3.403	0.182
	Yes	1	1.90%	5	9.40%	2	3.90%		
Scrotal Hematoma	No	53	98.10%	46	86.80%	46	90.20%	4.816	0.090
	Yes	1	1.90%	7	13.20%	5	9.80%		
Pelvic Seroma/Hematoma	No	52	96.30%	50	94.30%	51	100.00%	2.795	0.247
	Yes	2	3.70%	3	5.70%	0	0.00%		
Wound Seroma /Hematoma	No	53	98.10%	46	86.80%	42	82.40%	7.313	0.026*
	Yes	1	1.90%	7	13.20%	9	17.60%		
Wound Infection /Dehiscence	No	54	100.00%	49	92.50%	48	94.10%	3.973	0.137
	Yes	0	0.00%	4	7.50%	3	5.90%		
Hospital stay (Days): (mean ± SD) (Min – Max)		1.11 ± 0.32 1–2		1.77 ± 0.42 1–2		1.41 ± 0.50 1–2		33.81	<0.001*
Return to daily activity (Days): (mean ± SD) (Min – Max)		5.87 ± 0.97 5–8		10.64 ± 0.96 9–12		12.10 ± 1.02 11–15		578.02	<0.001*
Return to work (Days): (mean ± SD) (Min – Max)		12.30 ± 1.47 10–15		19.85 ± 1.06 18–22		20.20 ± 1.79 12–24		492.51	<0.001*

**Table 3:** Multivariate analysis of predictors for early postoperative complications.

		No early postop complications (n = 133)		Early postop complications (n = 25)		Odds Ratio	95% CI of OR		P
		N	%	N	%		Lower Bound	Upper Bound	
Smoking	Non smoker	21	15.80%	1	4.00%	24.452	1.074	556.549	0.045*
	Smoker	112	84.20%	24	96.00%				
Obesity	Non obese	116	87.20%	7	28.00%	0.149	0.028	0.787	0.025*
	Obese	17	12.80%	18	72.00%				
DM	No	120	90.20%	8	32.00%	0.187	0.033	1.059	0.058*
	Yes	13	9.80%	17	68.00%				
EHS Anatomical Classification	Bilateral Medial Hernia	74	55.60%	2	8.00%	3.139	1.389	7.096	0.006*
	One Medial and one Lateral Hernia	37	27.80%	7	28.00%				
	Bilateral Lateral Hernia	22	16.50%	16	64.00%				
EHS Size Classification	Size 2 both sides	64	48.10%	1	4.00%	0.06	0.005	0.682	0.023*
	Size 2 one side & size 3 other side	64	48.10%	9	36.00%				
	Size 3 both sides	5	3.80%	15	60.00%				
Surgery	Lap TAPP	51	38.30%	3	12.00%	0.023	0.001	0.369	0.008*
	Open PP Repair	42	31.60%	11	44.00%				
	Bilateral Lichtenstein	40	30.10%	11	44.00%

**Table 4:** Late outcomes: recurrence, QOL & patient's satisfaction after 3 years.

		Bilateral TAPP (n = 54)		Open PP Repair (n = 53)		Bilateral Lichtenstein (n = 51)		X2/F	P
		No	%	No	No	%	No		
Follow up (Months): (mean ± SD) (Min – Max)		35.19 ± 2.07 33–42		35.81 ± 1.72 32–42		36.05 ± 2.04 32–43		1.907	0.154
Recurrence Within 3 years	No	52	96.30%	49	92.50%	50	98.00%	2.018	0.365
	Yes	2	3.70%	4	7.50%	1	2.00%		
CCS^a^; Chronic pain	CCS 0 = No	48	88.80%	40	75.40%	33	64.70%	12.817	0.046*
	CCS 1–2 = Mild	5	9.30%	10	18.90%	12	23.50%		
	CCS 3–4 = Moderate	1	1.90%	3	5.70%	3	5.90%		
	CCS 5 = Severe	0	0.00%	0	0.00%	3	5.90%		
CCS; Mesh Sensation	CCS 0 = No	47	87.00%	38	71.70%	32	62.70%	10.78	0.029*
	CCS 1–2 = Mild	7	13.00%	12	22.60%	13	25.50%		
	CCS 3–4 = Moderate	0	0.00%	3	5.70%	6	11.80%		
	CCS 5 = Severe	0	0.00%	0	0.00%	0	0.00%		
CCS; Limitation of Movement	CCS 0 = No	51	94.40%	38	71.70%	39	76.40%	11.10	0.025*
	CCS 1–2 = Mild	2	3.70%	12	22.60%	11	21.60%		
	CCS 3–4 = Moderate	1	1.90%	3	5.70%	1	2.00%		
	CCS 5 = Severe	0	0.00%	0	0.00%	0	0.00%		
Total CCS score: (mean ± SD) (Min – Max)		10.04 ± 19.20 0–94		29.15 ± 25.10 0–96		32.86 ± 27.11 0–96		13.87	<0.001*
Patient satisfaction score	Very unsatisfied, Score 0-2	2	3.70%	5	9.40%	5	9.80%	51.53	0.001*
	Unsatisfied, Score 3-4	1	1.90%	5	9.40%	8	15.70%		
	Neutral, score 5-6	9	16.70%	17	32.10%	11	21.60%		
	Satisfied, Score 7-8	10	18.50%	23	43.40%	21	41.20%		
	Highly Satisfied, Score 9-10	32	59.30%	3	5.70%	6	11.80%		
Satisfaction score: (mean ± SD) (Min – Max)		8.39 ± 2.37 0–10		5.79 ± 2.35 0–10		5.80 ± 2.64 0–10		19.81	<0.001*

a: CCS; Carolina comfort scale.

### 4.2 Predictors for early postoperative complications (Table 3)

Multivariate analysis using logistic regression test revealed that smoking, obesity, DM, and anatomical type, size of hernia and the type of surgery were independent predictive factors for early postoperative complication.

### 4.3 Late outcomes: recurrence, QOL and patients satisfaction (Table 4)

Follow-up duration ranged from 32 to 43 months with no significant difference between the 3 groups regarding mean duration of follow-up. The 3-years recurrence rate of hernia was relatively lower after bilateral Lichtenstein repair (2%) compared to 3.7% and 7.5% after Lap TAPP and open PP group respectively but with no significant difference (p 0.36). According to CCS for assessment of QOL, the frequency of chronic groin pain was significantly lower in Lap TAPP group, compared to open PP repair and Bilateral Lichtenstein repair (11.2% compared to 24.6% and 35.3%, p 0.046). Similarly, the frequency of mesh sensation was significantly lower in Lap TAPP group, compared to open PP repair and Bilateral Lichtenstein repair (13% compared to 28.3% and 37.3%, p 0.038). Three patients (5.6%) complained of limitation of movement after Lap TAPP compared to 28.3% and 23.6% of patients after open PP repair and Bilateral Lichtenstein repair respectively; (p 0.025). Mean Carolina comfort score was significantly lower in Lap TAPP group compared to open PP group and Bilateral Lichtenstein group, (10.04 ± 19.20 compared to 29.15 ± 25.10 and 32.86 ± 27.11, p 0.001). Mean satisfaction rate was significantly higher in Lap TAPP compared to open PP and Lichtenstein groups (8.39 ± 2.37 compared to 5.79 ± 2.35 and 5.80 ± 2.64, p < 0.001).

## 5 Discussion

Inguinal hernias occur in about 1–5% of the general population and its repair is the commonest operation in general surgical practice [7]. With the introduction of the tension-free mesh repair, Lichtenstein repair, the recurrence rate of hernia declined as low as 1–4% and Lichtenstein repair became the gold standard of inguinal hernia repair [8]. The recent development of laparoscopic hernia repair techniques offered a new alternative to conventional open surgery with the potential benefits of less postoperative pain and faster recovery [9,10]. However, laparoscopic repair has been slow to gain acceptance, perhaps due to issues related to surgical technique, indications, learning curve and reported rare but serious complications [11]. It is estimated that about 8–30% of inguinal hernia patients have bilateral hernias [12]. Only few studies have discussed bilateral inguinal hernia, and the results were often mixed with those of unilateral or recurrent hernia [13,14]. The Hernia Surgery Group recommended a one stage mesh repair of bilateral inguinal hernia [15], however, the ideal approach is still controversial with no large studies available comparing different approaches of bilateral hernia repair. The aim of this randomized prospective study was to evaluate if laparoscopic TAPP repair offers advantages over open tension-free mesh repair of bilateral inguinal hernia.

In the current study, intraoperative bleeding happened in one patient (1.9%) in Lap TAPP and two patients (3.8%) in open PP repair group without the need for blood transfusion. The cause of bleeding in Lap TAPP group was injury of inferior epigastric artery while in open PP repair group, the source of bleeding was the perivesical fat. Laparoscopic TAPP repair had significantly longer operative time in the current study, compared to other groups. This may be explained by the more time needed for setup of laparoscopy also, where the contralateral repair by TAPP requires opening the peritoneum again, repairing the hernia defect and then closing both sides of the peritoneum incision. In his meta-analysis, Scheuermann et al. [16] reported a longer operative time of Lap TAPP in comparison to Lichtenstein repair. Hauters et al. [17], found no significant difference in operative time between Bilateral Lap TAPP and open PP repair while Nada et al. [18], reported shorter operative time of Bilateral Lap TAPP compared to open PP repair (83.6 ± 14.7 vs. 96.3 ± 19.7). In our study, Lichtenstein repair was significantly faster than open PP while, Malazgirt et al. [19] and Talha et al. [7], found that operative time of open PP repair was significantly shorter time than bilateral Lichtenstein repair because they didn't fix the mesh in open PP repair while we did.

Incidence of early postoperative complications was less after Laparoscopic TAPP repair (only 5.6%), compared to open mesh repair without difference between open PP and bilateral Lichtenstein groups. Scheuermann et al. [16] found no significant difference in frequency of postoperative complications in a meta-analysis comparing Lap TAPP and Lichtenstein repairs. Similarly, Nada et al. [17], found no significant difference in postoperative complications rates after Bilateral Lap TAPP and open PP repair. Talha et al. [7] and Malazgirt et al. [19], found no significant difference between open PP and bilateral Lichtenstein regarding postoperative complications.

Laparoscopic TAPP repair had superior immediate postoperative outcomes including significantly less postoperative pain scores, shorter duration of hospital stay, time till return to normal activity and to work. Many previous studies comparing laparoscopic versus open mesh repair in bilateral inguinal hernia revealed that laparoscopic bilateral inguinal hernia repair had significantly less postoperative pain with shorter hospital stay and earlier return to work [12,17,[20], [21], [22]]. Feliu et al. [13], found that about 60% of laparoscopic repairs were treated as day cases. These better outcomes are explained by the advantages of performing the two hernia repairs via the same three keyhole incisions [15]. In our study, there was no statistically significant difference between open PP and bilateral Lichtenstein regarding postoperative hospital stay, return to normal daily activities and work, similar result was reported by Talha et al. [7] and Malazgirt et al. [19].

After 3 years, there was no statistically significant difference in recurrence rates between the 3 groups although it was less in bilateral Lichtenstein repair group (only 2%). Regarding the 7 patients who developed recurrence, 2 recurrence in TAPP group occurred within 6 months of surgery and a missed indirect hernia was detected in the reoperation, the 4 recurrences after open PP repair were detected later (after 12 months) and were associated with development of postoperative pelvic seroma or hematoma, while the one recurrence after Lichtenstein repair was detected later (after 24 months) and corresponded to a direct defect. Nada et al. [18], reported no significant difference in recurrence rate after bilateral Lap TAPP compared to open PP repair.

According to CCS for assessment of QOL, the frequency of Chronic groin pain, mesh sensation and limitation of movement were significantly lower in Lap TAPP group, compared to open PP repair and Bilateral Lichtenstein repair. Previous meta-analyses described less chronic pain after laparoscopic versus open hernia repair [9,10,16]. In agreement with Talha et al. [7] and Malazgirt et al. [19] we found no significant difference between open PP and bilateral Lichtenstein regarding chronic groin pain.

Mean satisfaction rate was significantly higher in Lap TAPP compared to open PP and Lichtenstein groups with significantly higher frequency of patients very satisfied. Patients of lap group attributed their satisfaction to better cosmetic outcomes and postoperative course with less pain and early ambulation. Patients of open group attributed their dissatisfaction mainly to unsatisfactory cosmetic appearance of the wound and chronic groin pain. Similar data was found by Nada et al. [18], who reported significantly higher patients satisfaction after bilateral Lap TAPP compared to open PP repair.

## 6 Conclusion

One stage laparoscopic TAPP for uncomplicated primary bilateral inguinal hernia has superior early postoperative outcome, less chronic pain and higher patients' satisfaction rate compared to open approaches with accepted low recurrence rate.

## 7 Limitations

Firstly, patients in TAPP and open PP repair groups were operated under general anesthesia while in Lichtenstein group most patients were operated under spinal anesthesia. This means that patients with contraindication for general anesthesia may be treated by open PP repair or Bilateral Lichtenstein under spinal anesthesia while couldn't be offered laparoscopic hernia repair. Also, immediate postoperative pain score couldn't be compared between the 3 groups because of the bias related to analgesic effect of spinal anesthesia used mainly in the Lichtenstein group. Secondly, all patients in laparoscopic group were treated by TAPP although some guidelines recommend Totally Extra Peritoneal repair (TEP) in bilateral inguinal hernia repair. We have chosen TAPP not TEP because TAPP has the advantages of being easier to perform, possibility of standardization, shorter learning curve, and possibility of performing diagnostic laparoscopy [23], although it may be associated with significantly higher incidence of early postoperative pain, longer operative time, and cord edema, compared to TEP which may be associated with a significant higher incidence of seroma formation [24,25].

## 8 Recommendations

A larger multi-centers randomized, controlled trial including also the TEP laparoscopic approach and including patients with recurrent hernia and lower abdominal surgery is needed to define optimum inclusion and exclusion criteria for different approaches for bilateral inguinal hernia repair.

## Authors contributions

Mohamed M. Elmessiry: Study Conception and design: Acquisition, analysis and interpretation of data, Drafting and revising the article. Ahmed A. Gebaly: Acquisition, analysis and interpretation of data, Drafting and revising the article.

## Funding

None to declare.

## Ethical approval and registration

This study was approved by Alexandria Faculty of Medicine medical ethics committee (No: 0302765) and registered in ClinicalTrial.gov (NCT04437784). https://clinicaltrials.gov/ct2/show/NCT04437784?term=Mohamed+Elmessiry&draw=2&rank=2#wrapper.

## Provenance and peer review

Not commissioned externally peer reviewed.

## Consent

Informed written consent was taken from all patients before being included in this study.

## Registration for research details

1. Name of the registry: ClinicalTrials.gov.

2. Unique Identifying number or registration ID: NCT04437784.

3. Hyperlink to your specific registration (must be publicly accessible and will be checked).

## Declaration of competing interest

None to declare.
